# [Retracted] miR‑218 inhibits the migration and invasion of glioma U87 cells through the Slit2‑Robo1 pathway

**DOI:** 10.3892/ol.2023.14068

**Published:** 2023-09-25

**Authors:** Jian-Jun Gu, Guang-Zhong Gao, Shi-Ming Zhang

Oncol Lett 9: 1561–1566, 2015; DOI: 10.3892/ol.2015.2904

Following the publication of this article, a concerned reader has drawn to the attention of the Editorial Office that the data panels for the Transwell cell invasion assay data in Figs. 2A and 6A contained a number of overlaps, such that these data apparently originated from the same original sources where the results from differently performed experiments were intended to have been portrayed; moreover, certain of the scratch-wound assay data shown in Figs. 2C and 6C had already been submitted for publication in the following paper, written by different authors at different research institutes, albeit with a number of modifications [Shi Y, Wang Y, Luan W, Wang P, Tao T, Zhang J, Quan J, Liu N and You Y: Long non-coding RNA H19 promotes glioma cell invasion by deriving miR-675. PLoS One 23: 9, e86295].

Owing to the large number of overlapping data panels identified in comparing Figs. 2A and 6A, and the fact that the contentious data in Figs. 2C and 6C of the above article had already been published prior to its submission to *Oncology Letters*, the Editor has decided that this paper should be retracted from the Journal. After having been in contact with the authors, they accepted the decision to retract the paper. The Editor apologizes to the readership for any inconvenience caused.

